# Multipolar second harmonic generation in a symmetric nonlinear metamaterial

**DOI:** 10.1038/s41598-017-08039-1

**Published:** 2017-08-14

**Authors:** Omri Wolf, Salvatore Campione, Yuanmu Yang, Igal Brener

**Affiliations:** 1 Center for Integrated Nanotechnologies (CINT), Sandia National Laboratories, P.O. Box 5800, Albuquerque, NM 87185 USA; 2 Sandia National Laboratories, P.O. Box 5800, Albuquerque, NM 87185 USA

## Abstract

Optical nonlinearities are intimately related to the spatial symmetry of the nonlinear media. For example, the second order susceptibility vanishes for centrosymmetric materials under the dipole approximation. The latter concept has been naturally extended to the metamaterials’ realm, sometimes leading to the (erroneous) hypothesis that second harmonic (SH) generation is negligible in highly symmetric meta-atoms. In this work we aim to show that such symmetric meta-atoms can radiate SH light efficiently. In particular, we investigate in-plane centrosymmetric meta-atom designs where the approximation for meta-atoms breaks down. In a periodic array this building block allows us to control the directionality of the SH radiation. We conclude by showing that the use of symmetry considerations alone allows for the manipulation of the nonlinear multipolar response of a meta-atom, resulting in e.g. dipolar, quadrupolar, or multipolar emission on demand. This is because the size of the meta-atom is comparable with the free-space wavelength, thus invalidating the dipolar approximation for meta-atoms.

## Introduction

A key concept governing the efficiency of many nonlinear optical processes is modal overlap, i.e. how much similarity there is between the different eigenmodes of electromagnetic radiation that are interacting and exchanging energy^1^. It is therefore natural that such a concept was introduced in the field of nonlinear optical metamaterials, which has received significant attention in recent years^2–6^. There have been many attempts to design meta-atoms with optimal nonlinear conversion efficiencies^8^ and preferred directionality^8^. From this point forward, we restrict ourselves to a discussion regarding second harmonic (SH) generation, however the concepts introduced here may well be extended to other nonlinear processes.

In some cases^9^, the design paradigm was to investigate the linear response of the metasurface and increase the modal overlap between the electromagnetic modes resonating at the chosen fundamental frequency (FF) and the modes oscillating at the SH frequency of interest. This overlap integral, ${\rm{\Omega }}$, is of a form:

1 $${\rm{\Omega }}\propto \sum _{\begin{array}{c}i,j,k,\\ l,m,n\end{array}}\iiint {\chi }_{{\rm{kij}}}^{(2)}{{\rm{E}}}_{{\rm{i}}}^{{\rm{FF}},{\rm{m}}}{{\rm{E}}}_{{\rm{j}}}^{{\rm{FF}},{\rm{n}}}{{\rm{E}}}_{{\rm{k}}}^{{\rm{SH}},{\rm{l}}}{\rm{dV}}$$

where ${{\rm{\chi }}}^{(2)}$ is the second order nonlinear susceptibility tensor of the material and ${{\rm{E}}}_{{\rm{i}}}^{{\rm{FF}},{\rm{m}}},{{\rm{E}}}_{{\rm{j}}}^{{\rm{FF}},{\rm{n}}},{{\rm{E}}}_{{\rm{k}}}^{{\rm{SH}},{\rm{l}}}$ are the field profiles of modes m, n, l at the fundamental frequency (FF or ω) and second harmonic (SH or 2ω) frequency, respectively. The summation indexes i,j,k each span the three Cartesian directions and the integration is on the entire volume of the meta-atom unit cell. This overlap integral can be essentially understood as the overlap between the nonlinear polarization ${{\rm{P}}}_{{\rm{k}}}^{(2)}={{\rm{\chi }}}_{{\rm{kij}}}^{(2)}{{\rm{E}}}_{{\rm{i}}}^{{\rm{FF}},{\rm{m}}}{{\rm{E}}}_{{\rm{j}}}^{{\rm{FF}},{\rm{n}}}$, which drives the system at the SH frequency, and the electromagnetic modes supported by the meta-atom at the SH frequency.

To compute and optimize ${\rm{\Omega }}$ one needs the correct field profiles at both frequencies. These are often obtained from linear simulations with an external excitation (e.g. plane wave) at the frequencies of interest (FF and SH), where one invokes reciprocity in order to account for the fact that in the actual system, energy at the SH will be radiated out rather than injected. This is a good method to predict P^(2)^, but may fail in properly accounting for all the SH modes that have good overlap with the nonlinear polarization. The problem arises because this method only accounts for modes excited by the specific excitation pattern chosen (e.g. normal incidence plane-wave). This failure will be most substantial when the symmetry of the SH mode in question is different from the symmetry imposed by the chosen excitation scheme. In the first part of this work we investigate a case where this methodology fails; i.e. it cannot, in any practical implementation, correctly predict the SH generation of our structure.

In order to mitigate the flaw described above, many works chose to focus on metasurfaces with sub-wavelength periodicities^10–13^ which ensured that only eigenmodes that radiate in the normal direction determine the SH response.

Moreover, the analogy made between real atoms and meta-atoms implied that the dipole approximation^14^ (i.e. an approach that describes the interaction of atoms, molecules, and even solid-state systems with light when the wavelength is much larger than the average size of the coherent quantum state that is interacting with the radiation) could be readily translated to metamaterials in the form of a so-called ‘dipole-approximation for meta-atoms’ (DAMA). In the DAMA, the quantum states are replaced with the electromagnetic eigenmodes supported by a meta-atom (e.g., electric dipole resonance, magnetic quadrupole resonance, etc.), and since the meta-atoms are subwavelength then only the lowest order modes are considered. In general, higher order modes are not accounted for in the meta-atom response as they are assumed to radiate much less SH light.

In this work we argue that the DAMA may be insufficient to describe the behavior of nonlinear meta-atoms; this is because these meta-atoms are comparable in size to the relevant wavelength (in fact, meta-atoms are oftentimes larger than the wavelength in the constituent material). In other words, this means that treating meta-atoms as essentially dipole antennas is an over-simplification. While this argument also holds for linear systems, it is more important for nonlinear systems because in the latter case the SH ‘sources’ driving the system are distributed inside the antenna (as opposed to an external plane wave that normally drives a linear meta-atom). Additionally, the physical arrangement of these meta-atoms (or lattice constants) can be chosen at will; this freedom is important because it may allow modes with a null in the broadside direction to emit; this is in stark contrast to natural materials where the lattice constant is deeply sub-wavelength and no diffraction orders exist. We observe that by correctly accounting for symmetry considerations one can control the nonlinear radiation pattern of the meta-atoms. We use nonlinear finite-difference time-domain (FDTD) simulations^15^ followed by multipolar decompositions of the far-field radiation^14, 16, 17^ in order to analyze the nonlinear response of several meta-atoms.

**Figure 1:**
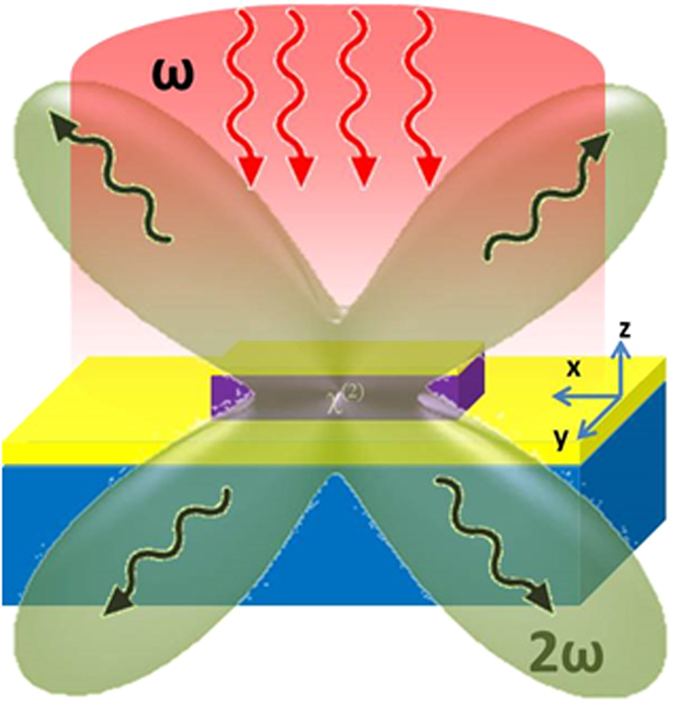
Schematic representation of the structure investigated in the first part: the red illumination represents the plane-wave excitation used and the green emission pattern represents the multipolar radiation at the second harmonic (SH) frequency. Dielectric cuboid dimensions *x*, *y*, *z*: 0.7 µm × 0.22 µm × 0.18 µm. Top metal thickness 40 nm. Partial gold backplane thickness 110 nm.

We start by investigating the structure shown in Fig. 1 consisting of a rectangular cuboid of nonlinear material with a metallic film on top, surrounded by a partial metallic back plane (the metal does not extend below the cuboid). This structure has not been optimized to maximize the SH conversion efficiency; the investigated design supports a near-perfect absorption resonance at the fundamental frequency, which should enhance the SH conversion efficiency.

The model we use for the material’s nonlinear susceptibility has a single non-vanishing term in the χ^(2)^ tensor at the *zzz* position (see supporting information for details). This simple tensor structure allows us to intuitively visualize the symmetry properties that are under investigation in this work. Furthermore, it is a good approximation of the nonlinearity associated with several materials such as lithium niobate and other ferroelectrics where one tensor element dominates, and an accurate description of the resonant nonlinearity that may arise from heterostructured semiconductors^18^. We note that the conclusions we draw from examining this simple nonlinearity model can be readily extended to other material systems where the symmetry of the χ^(2)^ tensor is more complicated. The structure in Fig. 1 exhibits a high degree of symmetry (2 reflection planes and a C2 axis); thus, the overlap integral (Eq. 1) involving the in-plane, cross-polarized, dipole modes conventionally used to enhance SH generation vanishes. Since the main point of this work is to demonstrate the possibility of SHG from a structure with C2 symmetry by leveraging the higher order modes at the SH frequency, we have not attempted to achieve a large SH conversion efficiency.

**Figure 2:**
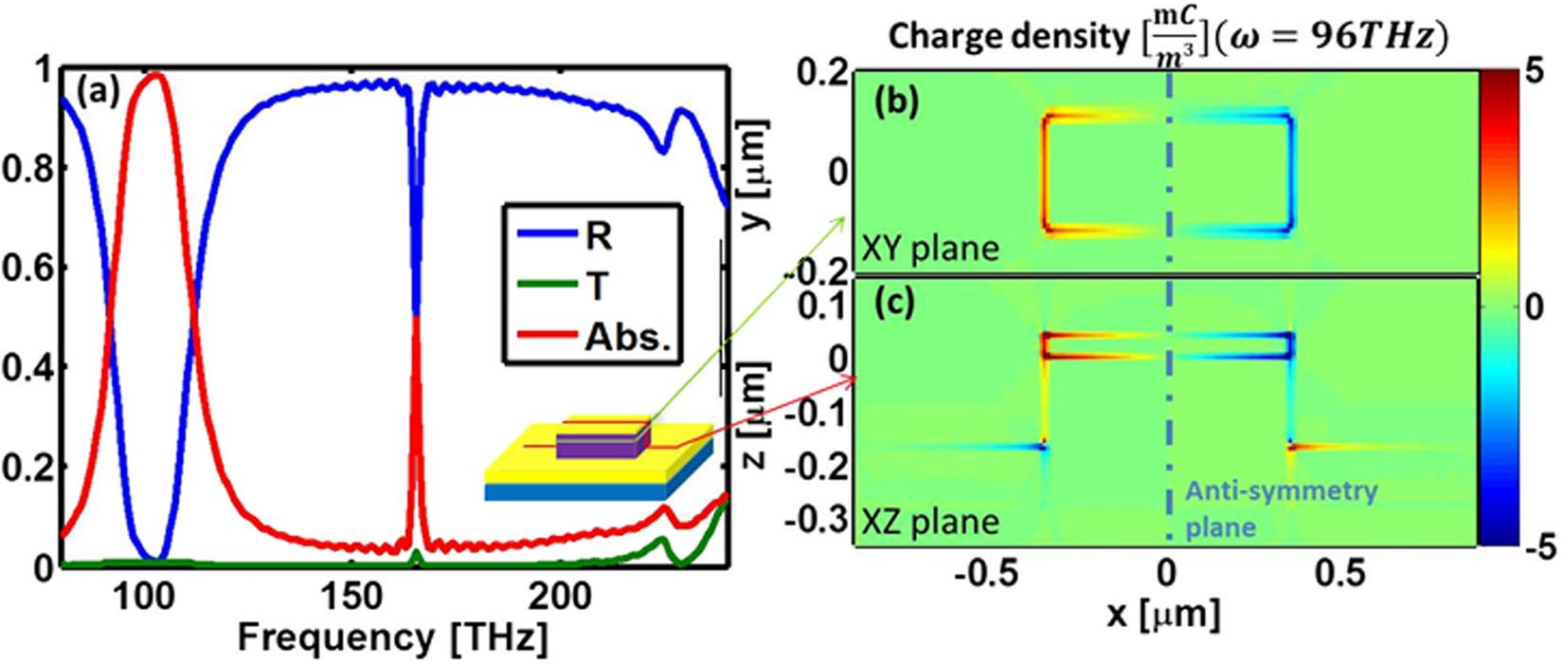
Linear response. (**a**) Linear characterization of an array of cuboid resonators: reflection (R), transmission (T), and absorption (Abs.). (inset) Schematic of the structure with colored lines denoting the locations of the slices of panels (**b**) and (**c**). (**b**,**c**) Total charge density oscillating at the fundamental frequency. The axes correspond to Fig. 1. The anti-symmetry plane created by the excitation is denoted with the vertical, blue dot-dash line.

The linear response of the cuboid in a periodic array (periods: *x, y* = 1.8 µm × 1 µm) is shown in Fig. 2(a). The excitation is a broadband, normal incidence plane-wave polarized along the long axis of the cuboid (*x*-axis). The structure exhibits near-perfect absorption for frequencies around the FF of interest (~100 THz); such high absorption has been shown to increase the SH conversion efficiency. No significant features appear at the corresponding SH frequency (~200 THz). This simulation suggests that our excitation scheme (i.e. normal-incidence plane-wave) is not coupling efficiently to any mode supported by the resonator at the SH frequency. The sharp feature at ~160 THz arises from the lateral periodicity of the structure, but this particular frequency is of no interest in this work (see the supporting information for the linear response to a y-polarized excitation). While our design is based on ref. 19, the essence of the results and conclusions in this work are independent of frequency.

Figure 2(b,c) depict the spatial profile of the total charge density,$\,\rho$, oscillating at the FF (calculated using Gauss’s law from the electric fields, ***E***, $\rho ={\varepsilon }_{0}\nabla \cdot {\boldsymbol{E}}$). Figure 2(b) portrays a slice along the XY plane 20 nm below the top metal while Fig. 2(c) depicts a slice through the center of the cuboid resonator in the XZ plane (axis orientations in Fig. 1). The anti-symmetry with respect to the YZ plane (dot-dash line) is clearly visible; it is dictated by the symmetry of the excitation adopted in the simulation.

**Figure 3:**
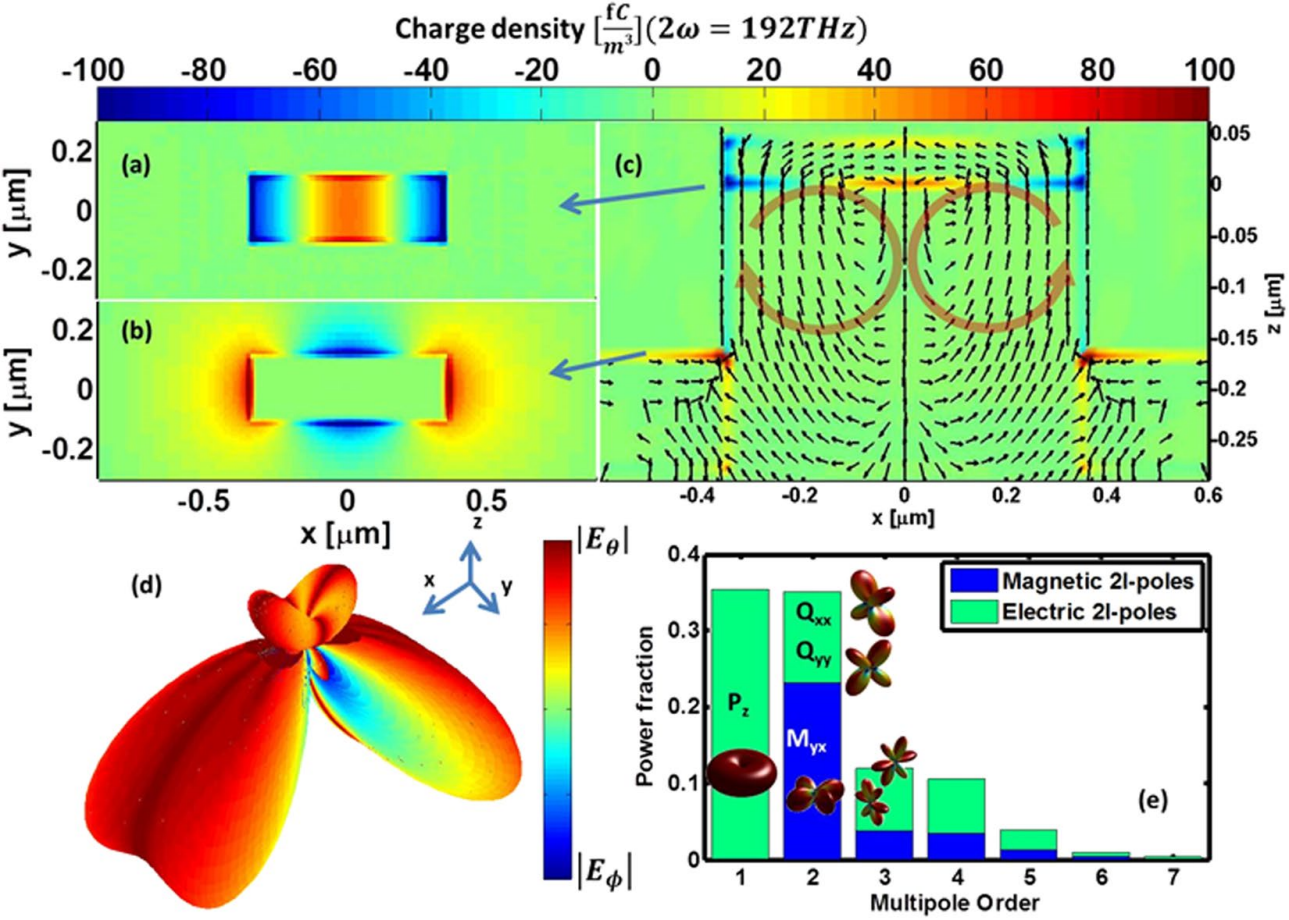
Isolated resonator nonlinear response. (**a–c**) Total charge density distributions oscillating at the SH frequency. (**a**) and (**b**) are XY plane slices where the z position is denoted by the blue arrows. (**c**) Slice position same as Fig. 2(c). The black arrows denote the direction of the total current density. The red circulating arrows highlight the current trends responsible for the lowest non-dipolar mode. (**d**) Far field radiation profile. The unit vectors are given for orientation. (**e**) Fraction of power radiated as a function of multipole order. The main contributing moments are explicitly named and their radiation pattern is schematically given.

We now turn to the nonlinear response of an isolated meta-atom. Here we use narrow band plane wave excitation at the FF with a central frequency at ~100 THz with a spectral bandwidth with a FWHM of 0.44 THz and examine the response at the SH frequency; no energy is injected into the simulation at the SH frequency (more details about the nonlinear simulation can be found in the supporting information). Figure 3(a–c) depict $\rho$ that oscillates in the structure at the SH frequency. Figure 3(a) is a slice (in the XY plane) through the bottom face of the metal bar, Fig. 3(b) is a slice through the top face of the partial backplane and Fig. 3(c) is a slice in the same position as Fig. 2(c). The blue arrows mark the z positions from which the horizontal slices (Fig. 3(a,b)) are taken. The black arrows denote the direction of the instantaneous total electric current (at the arrow origin) as calculated using Ampere’s law. From Fig. 3(a–c) it is clear that $\rho$ at the SH has a complicated distribution that requires high moments to describe. Furthermore, due to the nature of the second order nonlinearity ($\propto {{\boldsymbol{E}}}^{2}$) the symmetry of $\rho$ (i.e. symmetric with respect to the YZ plane) is opposite compared to the FF drive (e.g. compare Figs 2(c) and 3(c)).

To quantify the various radiating moments we record the electromagnetic fields outside of the cuboid, and expand them into a far-field radiation pattern (see supporting information for details). This is shown in Fig. 3(d): the radiation intensity, ${|{E}_{\theta }|}^{2}+{|{E}_{\phi }|}^{2}$, is represented by the distance of a point from the origin while partial polarization information, $\arctan (|{E}_{\theta }|/|{E}_{\phi }|)$, is represented by the color of the point (this plot does not distinguish between certain linear and circular polarizations). First we note the null along the *z* axis: this null is a direct result of the symmetry properties of our structure. This result confirms that if one were to compute ${\rm{\Omega }}$ using only normal incidence excitation one would predict no SH response from this resonator, a conclusion which is obviously wrong.

Figure 3(e) presents the multipole decomposition for the radiation pattern of the meta-atom (details of the decomposition procedure can be found in the supplemental information). We plot the fraction of the total power in each multipolar moment as a function of their multipole order. The leading contributions are explicitly named together with their radiation patterns. As expected, the *z*-polarized dipole is significant while the forbidden, in-plane dipoles are absent. We also note significant contributions from high-order moments. A heuristic relation between $\rho$ and the multipole decomposition is given in the supplemental information.

Attempting to reproduce these results using the overlap integral methodology is essentially impossible because the excitation scheme required to correctly account for the relative power of all the radiating modes requires *guessing* the radiation pattern from Fig. 3(d) or at least creating an excitation scheme similar to it. In this case one would need at least 10 (the number of lobes in Fig. 3(d)) plane-wave sources incident from different angles (other more complicated excitation schemes could also be used, but again these require some knowledge of where to locate the sources).

**Figure 4:**
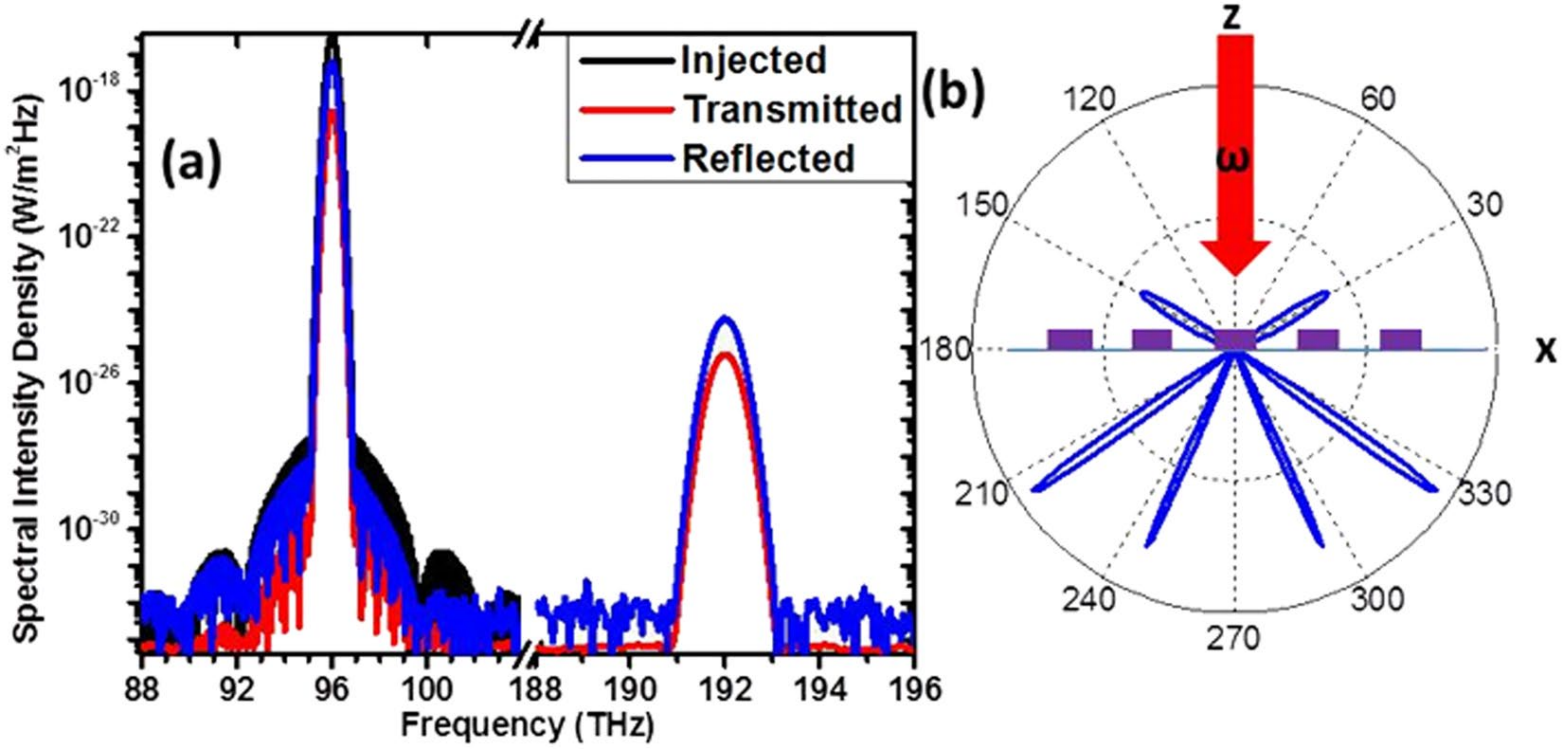
Array nonlinear response. (**a**) Spectral intensity density as injected and observed. (**b**) Far field cross section of the SH signal in the XZ plane: the plane wave excitation direction (in air) is marked with the red arrow. The far-field response is calculated with a 10 × 10 µm aperture, and the purple rectangles represent the metasurface sitting on the substrate.

Next we examine the nonlinear response of a periodic array. Figure 4(a) depicts the spectral intensity density injected into a simulation of a periodic meta-surface using a normal incidence plane-wave and the measured intensity in monitors far from the resonator array both on the air side (reflected) and the substrate side (transmitted). It is clear that SH radiation is being radiated into the far field at 192 THz. This structure has similar conversion efficiencies (defined as the ratio between the SH radiated intensity and the squared excitation intensity) compared to “traditional” structures such as split ring or T resonators as were used in refs 4, 9, however further investigations into this aspect are outside of the scope of this work.

In Fig. 4(b), we show a cross section of the far field radiation from the array. In antenna theory it is shown that the far-field of an array can be decomposed into the far-field of a single unit cell multiplied by an *array-factor* related to the periodicity^20^. The *array-factor* has maxima at ${\theta }_{peak}$ which follow the relation $sin\,{\theta }_{peak}=n\lambda /2d$ where *λ* is the wavelength in the medium, *d* is the spacing and *n* is an integer called the diffraction order. This implies that the normal direction (${\theta }_{peak}=0\,$) will always have a peak, the 0^th^ diffraction order (unless the single resonator has a null there). However, in the figure we see that this 0^th^ order is missing both in reflection and transmission. This confirms that the active modes (i.e. the higher-order modes) indeed cannot couple to a normal incidence plane-wave. The first diffraction order, seen in reflection, matches the expected angle due to the period (1.8 µm) and wavelength (1.5 µm); and so do the diffraction orders on the transmission side when accounting for the refractive index of the substrate (*n* = *2.12*). In this numerical simulation, we show that one can control the directionality of the SH radiation from the metasurface through the periodicity, while using *identical* and *symmetric* meta-atoms.

We have shown that higher order modes may radiate more energy than lower order ones, a property that is in contrast with electrostatics where the potentials of higher order multipoles decay faster than lower order terms. We have also shown that the overlap integral method is severely limited in predicting the nonlinear response of certain structures that may be attractive for efficient nonlinear conversion. Next we discuss an approach for analyzing the nonlinear response that relies on fundamental symmetry considerations and is therefore very robust.

**Figure 5:**
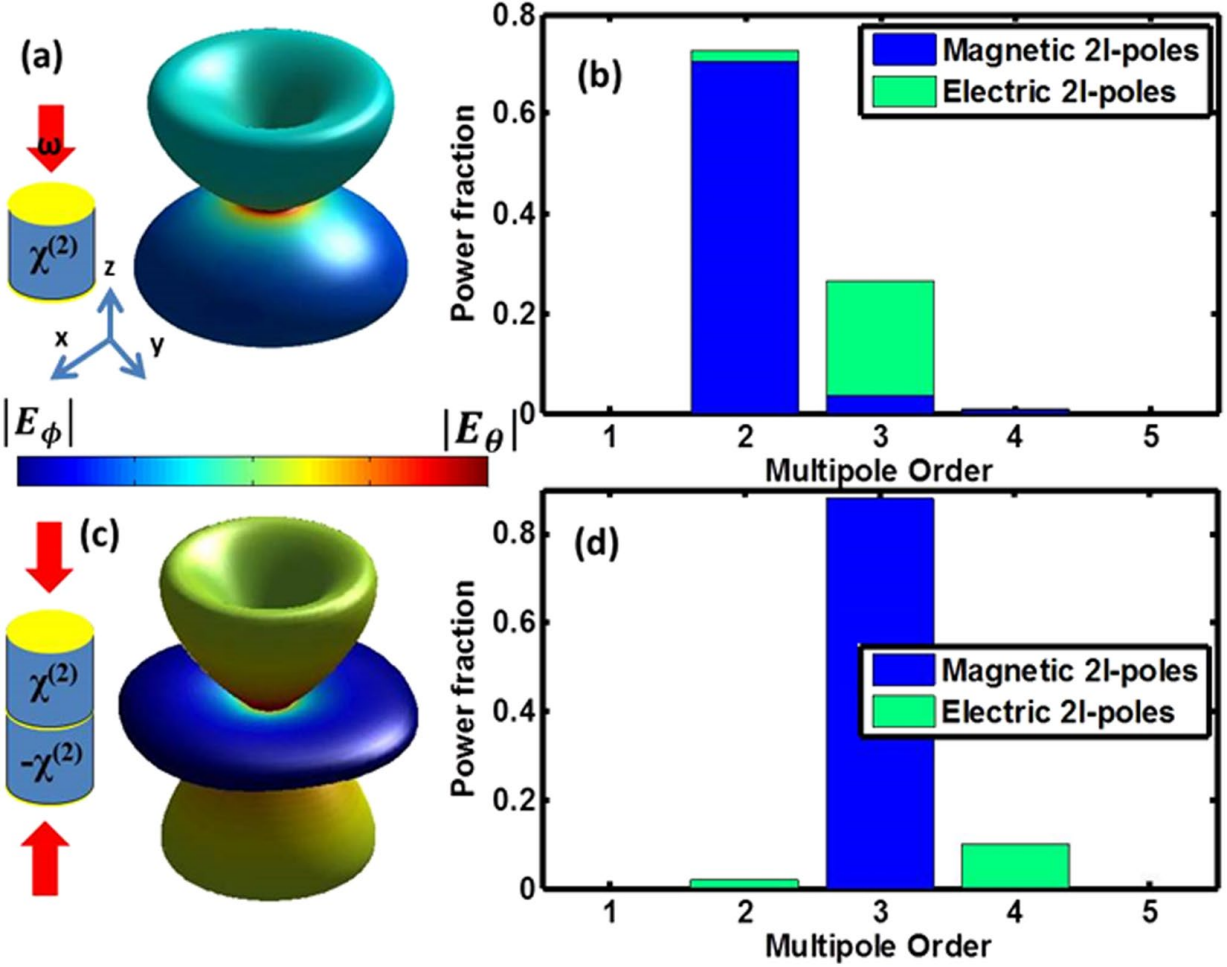
(**a**) Centro-symmetric resonator and the SH radiation pattern it produces. (**b**) Multipolar decomposition of the radiation pattern in (**a**). (**c**) Anti-centro-symmetric resonator and the SH radiation pattern it produces. (**c**) Multipolar decomposition of the radiation pattern in (**b**). (**d**) Multipolar decomposition of the radiation pattern in (**c**). Cylinder radius: 0.35 µm, height: 0.4 µm, Gold disk height: 20 nm.

Since each multipole order can be associated with a certain symmetry (e.g. odd orders are antisymmetric while even orders are symmetric) one could design a structure and excitation scheme in which the lowest radiating multipole at the SH can be chosen at will. To illustrate this point we design two meta-atoms with higher symmetries: first, a fully centrosymmetric meta-atom (see inset to Fig. 5(a)), namely a cylinder with a thin metal film on the two faces. We repeat the single resonator simulation discussed above, but this time we use circular polarization (in order to maintain an axial symmetry) at the FF to excite the resonator. Figure 5(a) depicts the SH far-field for the cylindrical resonator, with the inset reporting a schematic of the structure. Figure 5(b) depicts the multipolar decomposition; as designed, there is no dipolar component to the SH radiation. Linearly polarized excitation will redistribute power between different quadrupoles.

To inhibit the quadrupolar response we need to create two identical quadrupoles that radiate with a π phase shift. In Fig. 5(c) we show the radiation pattern of a meta-atom composed of two stacked cylinders from the previous case (see inset to Fig. 5(c)); in order to create the phase difference, the *χ* ^(2)^ is set to have opposite signs in the two sub-cylinders (this could be done for example, using periodic polling^21^ in nonlinear crystals). In addition, we use two counter-propagating, circularly polarized plane-waves as the FF excitation. Figure 5(d) depicts the calculated multipolar decomposition; as expected, the quadrupolar term is negligible. This meta-atom is also centrosymmetric so the dipolar term is forbidden as well. It is also interesting to note that different lobes have different linear polarizations. Finally, we note that the dimensions of the structures shown in Fig. 5 were not optimized; these can be used to improve the FF resonant response and to control the relative power in the available modes at the SH. The basic symmetry described here guarantees that the forbidden modes won’t appear in the overall SH radiation.

By examining the second order nonlinear response of a highly symmetric, hybrid metal-dielectric nonlinear metamaterial, we show that the DAMA breaks down and significant second harmonic light is radiated into the far-field. Examination of the radiation pattern, charge, and current distributions reveal that more than 60% of the SH radiation is emitted in high multipole orders (with order 4 radiating about 8% of the power). Since the size of these meta-atoms is larger than the effective wavelength in them (but still smaller than the free-space wavelength), it is not surprising that they support charge oscillations in high multipolar orders. This phenomenon is rarely encountered in natural materials because there the emitter size (i.e. the mean volume of a coherent charge oscillation) is usually on the atomic or molecular length scale. We further show that the multipolar response (and through it the directionality and polarization) of a nonlinear meta-atom can be largely controlled by correctly accounting for the symmetry of the structure, the excitation, and the *χ* ^(2)^ tensor. A key idea here is that even-order nonlinear processes are symmetrizing (i.e. a spatially anti-symmetric excitation will result in a symmetric nonlinear polarization). This implies that high overlap integrals will more likely involve modes where the fundamental one is antisymmetric while the SH one is symmetric.

One immediate application of this work is towards maximization of nonlinear conversion efficiencies from metamaterial systems. The ability to take advantage of the higher order modes should, in principle, allow one to greatly boost the overlap integral. Furthermore, these modes may enhance control over emission directionality through, for example, correctly balancing the power in different multipoles (this is known as the generalized Kerker conditions^22^) or by arranging different multipolar meta-atoms in a super-cell. Another attribute of high order multipoles is that the photons associated with them carry high angular momentum. The latter can be used to efficiently excite transitions that are ordinarily weak due to angular momentum conservation.

### Data availability

All data generated or analysed during this study are included in this published article (and its Supplementary Information file).

## Electronic supplementary material

Supplementary Information
