# Supplementary material for: Multipolar second harmonic generation in a symmetric nonlinear metamaterial
Source: Sci Rep. 2017 Aug 14;7:8101. doi: 10.1038/s41598-017-08039-1 (PMC5556125; doi:10.1038/s41598-017-08039-1)
Supplement: Supplementary file 1 — Supplementary Information [file 41598_2017_8039_MOESM1_ESM.pdf]

Supplementary Information for "Multipolar second harmonic generation in a symmetric nonlinear metamaterial"

Omri Wolf[1,2], Salvatore Campione[2], Yuanmu Yang[1,2] and Igal Brener[1,2]

[1] Center for Integrated Nanotechnologies (CINT), Sandia National Laboratories, P.O. Box 5800, Albuquerque, NM 87185, USA

[2] Sandia National Laboratories, P.O. Box 5800, Albuquerque, NM 87185, USA

Correspondence to I.B. (ibrener@sandia.gov)

This document provides supplementary information to the manuscript "Multipolar second harmonic generation in a symmetric nonlinear metamaterial". It describes: the permittivity models used in the simulations, the linear response to Y-polarized light, additional details about the nonlinear FDTD simulations, extraction of the nonlinear polarization, near to far field expansion and multipolar decomposition and a heuristic analysis of the cuboid structure.

Permittivity model

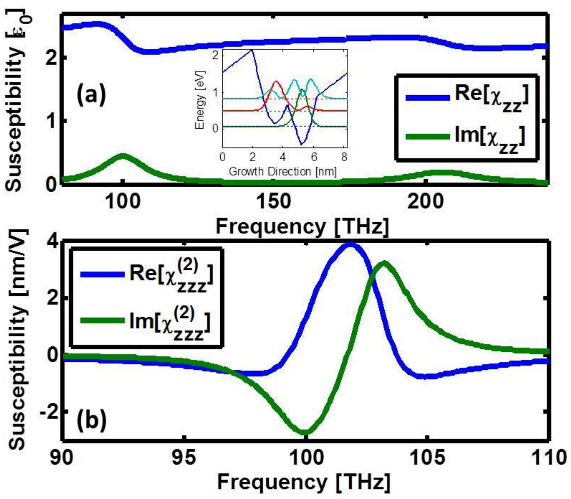

Figure S1(a) Linear susceptibility for the quantum wells (QWs) used in this study; it is based on QW intersubband absorption. The inset shows a typical band-structure of AlGaN QWs as used in the simulation. The blue curve represents the conduction band while the green, red, and cyan lines denote the relevant quantized subbands. (b) Dispersion of the second order nonlinear susceptibility responsible for second harmonic generation in QWs with doubly resonant intersubband transitions.

We use Intersubband transitions in Aluminum Gallium Nitride coupled QWs; the same model can be applied for other QW systems with intersubband transitions. The linear portion of the susceptibility does not have a significant effect on the results discussed in this work. A typical band structure diagram is given in the inset of Fig. S1(a). The linear susceptibility of such a system can be described by a diagonal permittivity tensor where the zz component has Lorentzian oscillators with center frequencies corresponding to the intersubband transition energies, as plotted in Fig. S1(a) (see [19] for formulas). It has been shown that these systems can be tailored to significantly enhance SH generation. This can be achieved by designing the QWs to have three sub-bands that are: (1) equally spaced in energy, (2) have significant transition dipole moments, and (3) have a high carrier concentration in the ground sub-band and nearly empty excited sub-bands. Similar to the linear part, the second order

nonlinear susceptibility tensor ($\chi^{(2)}$) describing this system has a single non-vanishing component in the zzz term (see [19] for formulas) this tensor form is also a good approximation of many nonlinear natural materials. In contrast to commonly used bulk and surface nonlinearities in conventional crystals, this nonlinearity is resonant and therefore very dispersive. Figure S1(b) depicts the calculated value of $\chi^{(2)}_{zzz}(\omega \rightarrow 2\omega)$ for our system. We acknowledge that our $\chi^{(2)}$ does not account for the nonlinearity arising from the bulk or the surface; however the value we use (~1 nm/V) for the intersubband nonlinearity (which is in accordance with modest experimental data) is one to two orders of magnitude larger than typically encountered values in bulk systems. This allows us to safely neglect all nonlinear contributions not related to the intersubband system. The expression used to calculate $\chi^{(2)}$ given in the plot is based on[9], and the parameters we use are similar to the experimental data in [19]. The AlN substrate is purely dielectric in the frequency range of interest to us and the metal films are assumed to be gold.

Linear response of the cuboid structure for Y polarized excitation:

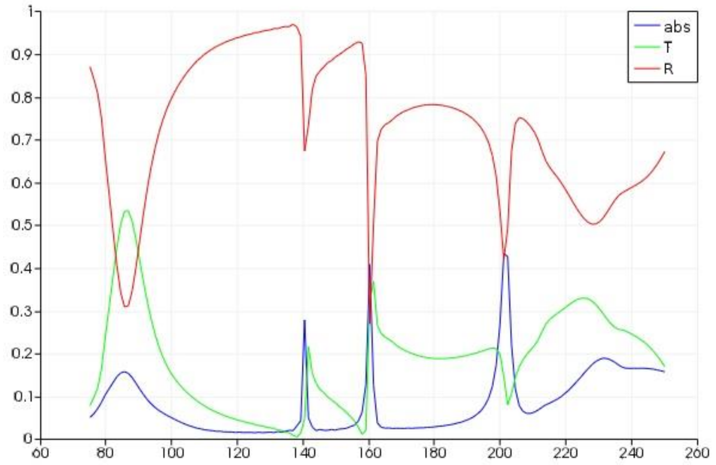

Figure S2 Transmission, reflection and absorption of the structure under *y*-polarized, broad-band, plane-wave excitation.

The absorption feature that appears at the SH frequency (in figure S2) is attributed to a mode that cannot be coupled into by the nonlinear polarization due to symmetry considerations. This can be visualized by examining the integral in Ω along the y-axis. It involves integrating the electric fields oscillating at the SH (Fig S3-bottom) multiplied by the nonlinear polarization $P^{(2)}$ (figure S4(a)); the former is antisymmetric while the latter is symmetric so the total integral will be zero.

Figure S4(a,b) depict the calculated nonlinear polarization $P^{(2)}$ at the SH. This is computed by squaring the z-component of the electric field at the FF inside the semiconductor. As expected, the spatial distribution of $P^{(2)}$ is symmetric with respect to the YZ plane; this implies that it can't drive a dipolar mode polarized along the x-axis (since dipolar modes are anti-symmetric).

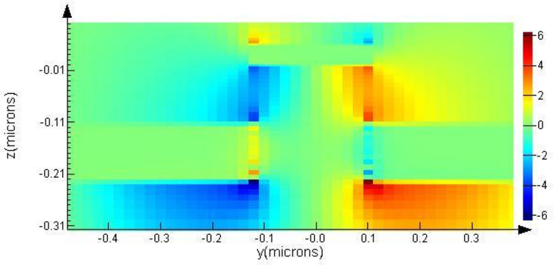

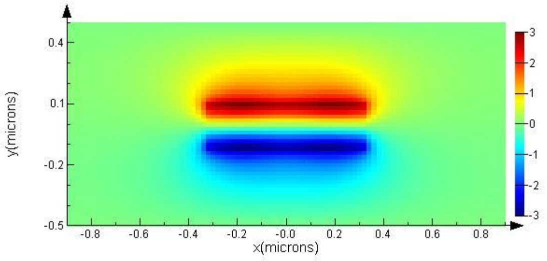

Figure S3. Linear response to *y*-polarized excitation. (Left) The *z*-component of the electric field in the YZ plane through the resonator center (*x* = 0). (Right) The *z*-component of the electric field in the XY plane 20 nm under the top metal bar.

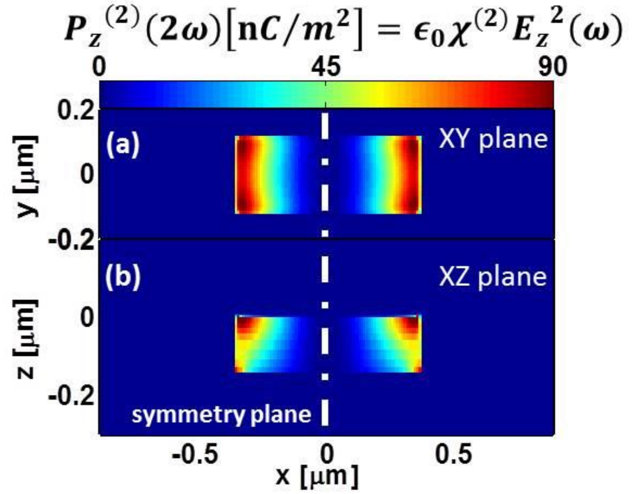

Figure S4. The *z*-component of the nonlinear polarization at the SH frequency. Slice position in (a) and (b) is the same as in Fig. 2(b) and (c) of the main text, respectively.

Additional details about the nonlinear FDTD simulations

As discussed above we account only for $\chi^{(2)}$ arising from the intersubband transitions. We use narrow band excitation (at 96 THz with ~0.3 THz bandwidth) and therefore consider $\chi^{(2)}$ to be nondispersive in the frequencies of interest using the values reported in Fig. 1(c).

For the isolated resonator simulations we avoid edge effects by increasing the simulation's lateral dimensions and we verify that the $P^{(2)}$ profile in this simulation matches the $P^{(2)}$ profile in an array simulation.

The spectral power of the excitation plane-wave at the SH is orders of magnitude weaker than the SH power generated by the meta-atom so a 'total-field-scattered-field' approach is not necessary.

The nonlinearity is implemented by adding a term to the polarization: $P(t) = P_{linear}(t) + \varepsilon_0\chi^{(2)}E^2(t)$

$P^{(2)}$ extraction from nonlinear single resonator simulation

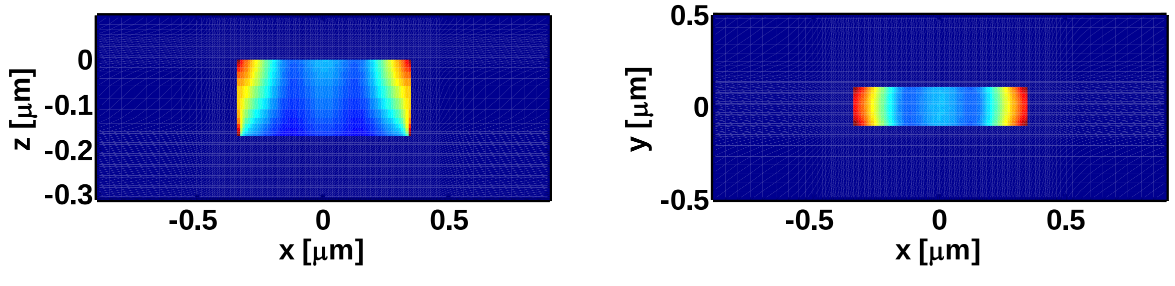

Figure S5. Absolute value of the z component of the nonlinear polarization at the SH frequency as extracted from the electro-magnetic field output of the nonlinear simulation of the single resonator, (left) slice in the XZ plane, (right) slice in the XY plane.

In the metal, the current arises from the movement of free charges, while in the semiconductor it is bound current J_b. In a material with no magnetization (relative permeability equal to one) the bound current is equal to the temporal change (time derivative) of the polarization; in the semiconductor the polarization at the SH frequency has two components: the linear $P^{(1)}$ and nonlinear $P^{(2)}$ parts. Since $J_b = \frac{\partial P}{\partial t} = \frac{\partial P^{(1)}}{\partial t} + \frac{\partial P^{(2)}}{\partial t}$ and $P^{(1)}$ is proportional to the electric field $P^{(1)} = \varepsilon E$, we can extract

$P^{(2)}$ to confirm that the nonlinear polarization in the single resonator simulation (Fig. S5) and the array simulation (Fig. S4) are similar.

Near to far-field expansion and multipolar decomposition details

The recorded near-fields are used as sources for far-field radiation measured on a sphere with a radius of 1 m. For the cuboid resonator we correct the field amplitudes in the bottom half space to account for the fact that these fields are propagating inside a dielectric.

To compute the multipolar decomposition we first normalize the total power in the far-field pattern to one. We then compute the l, m coefficient by calculating the inner product of a vector spherical harmonic (VSH) of order l and degree m and the normalized far-field radiation pattern. The normalization used for the VSHs is:

$$\mathbf{\Psi}_l^m * \mathbf{\Psi}_{l'}^{m'} = \delta_{ll'}\delta_{mm'}$$
$$\mathbf{\Phi}_l^m * \mathbf{\Phi}_{l'}^{m'} = \delta_{ll'}\delta_{mm'}$$
$$\mathbf{\Phi}_l^m * \mathbf{\Psi}_{l'}^{m'} = 0$$

where $\mathbf{\Psi}_l^m = r\nabla Y_l^m$ is the "longitudinal polarized" VSH, $\mathbf{\Phi}_l^m = \boldsymbol{r} \times \nabla Y_l^m$ is the "azimuthally polarized" VSH, $\delta_{ll'}$ is the kroneker delta and * denotes inner product, Y_l^m are the scaler spherical harmonics. This procedure ensures that the squares of the coefficients in the expansion represent the fraction of power radiating in each multipole.

The radially polarized VSHs ($Y_l^m \hat{\boldsymbol{r}}$) are not used because the far field radiation has only transverse electromagnetic fields. We associate $\mathbf{\Psi}_l^m$ with 'electric-multipoles' and $\mathbf{\Phi}_l^m$ with 'magnetic-multipoles'.

For clarity in Fig 4(e), 5(b), 5(d) we sum over moments with different degrees (m) within an order (l) but distinguish between magnetic- and electric- moments.

Multipolar decomposition of cuboid structure - heuristic analysis

Most of the radiation is directed into the high index substrate as can be expected from an embedded "emitter" at an interface. We see that about a third of the power is radiated from a z-oriented electric dipole (P_z); due to the in-plane symmetry of the structure only the z-oriented dipoles are allowed. the linearly polarized excitation scheme chosen does not allow the magnetic z-dipole to be excited; when looking at the charge distribution depicted in Fig. 3(c) one can visualize two in-phase dipoles along the vertical edges of the resonator: these are only partially cancelled by the additional dipoles visible along the long edges of the resonator in Fig. 3(a,b). We can attribute the strength of this mode to the strong asymmetry (in the z direction) in the spatial profile of $P^{(2)}$ as can be seen in Fig. S4(a). The next strongest radiating moment is a magnetic quadrupole mode (M_{yx}) with about 20% of the power: the generator of this mode may be seen in the current distribution (arrows in Fig. 3(c)) as two counter-circulating loops. The fact that these current loops are far from circular gives rise to the higher order (but much weaker) magnetic moments. The in-plane electric quadrupoles (Q_{xx} and Q_{yy}) that radiate about 10% of the power may be related to the in-plane charges seen in Fig. 3 (a,b): these diagonal terms of the quadrupole tensor receive large contributions from symmetric (along the named axis) charge distributions which are clearly visible in the figures. Two electric octapoles also contribute about 8% power and most likely arise from the relative spatial arrangement of the two electric quadrupoles discussed above.
